# Daily Liquorice Consumption for Two Weeks Increases Augmentation Index and Central Systolic and Diastolic Blood Pressure

**DOI:** 10.1371/journal.pone.0105607

**Published:** 2014-08-25

**Authors:** Miia H. Leskinen, Elina J. Hautaniemi, Anna M. Tahvanainen, Jenni K. Koskela, Marika Päällysaho, Antti J. Tikkakoski, Mika Kähönen, Tiit Kööbi, Onni Niemelä, Jukka Mustonen, Ilkka H. Pörsti

**Affiliations:** 1 Department of Internal Medicine, School of Medicine, University of Tampere, Tampere, Finland; 2 Department of Clinical Physiology, Tampere University Hospital, Tampere, Finland; 3 Department of Laboratory Medicine and Medical Research Unit, Seinäjoki Central Hospital, Seinäjoki, Finland; 4 Department of Internal Medicine, Tampere University Hospital, Tampere, Finland; Kurume University School of Medicine, Japan

## Abstract

**Background:**

Liquorice ingestion often elevates blood pressure, but the detailed haemodynamic alterations are unknown. We studied haemodynamic changes induced by liquorice consumption in 20 subjects versus 30 controls with average blood pressures of 120/68 and 116/64 mmHg, respectively.

**Methods:**

Haemodynamic variables were measured in supine position before and after two weeks of liquorice consumption (daily glycyrrhizin dose 290–370 mg) with tonometric recording of radial blood pressure, pulse wave analysis, and whole-body impedance cardiography. Thirty age-matched healthy subjects maintaining their normal diet were studied as controls.

**Results:**

Two weeks of liquorice ingestion elevated peripheral and central systolic and diastolic blood pressure (by 7/4 and 8/4 mmHg, 95% confidence intervals [CI] 2-11/1-8 and 3-13/1-8, respectively, *P*<0.05), and increased extracellular volume by 0.5 litres (*P*<0.05 versus controls). Also augmentation index adjusted to heart rate 75/min (from 7% to 11%, 95% CI for change 0.3-7.5, *P*<0.05) and aortic pulse pressure (by 4 mmHg, 95% CI 1-7, *P*<0.05) were elevated indicating increased wave reflection from the periphery. In contrast, peripheral (−3/−0.3 mmHg) and central blood pressure (−2/−0.5 mmHg), aortic pulse pressure (−1 mmHg), and augmentation index adjusted to heart rate 75/min (from 9% to 7%) decreased numerically but not statistically significantly without changes in extracellular volume in the control group. Heart rate, systemic vascular resistance, cardiac output, and pulse wave velocity did not differ between the groups.

**Conclusions:**

Two weeks of daily liquorice consumption increased extracellular volume, amplified pressure wave reflection from the periphery, and elevated central systolic and diastolic blood pressure.

**Trial Registration:**

EU Clinical Trials Register EudraCT 2006-002065-39</url>

ClinicalTrials.gov NCT01742702

## Introduction

During recent years there has been a growing research interest concerning the role of mineralocorticoids and the mineralocorticoid receptor (MR) in hypertension [1]. MRs are found in both classical aldosterone target tissues (kidneys, colon and salivary glands), but also in non-classical target tissues like the heart, vascular wall, and the central nervous system [2].

Both aldosterone and cortisol can bind to the MR with comparable affinity *in vitro* [3], [4]. However, normally only aldosterone acts as the physiologic agonist of the MR [3], [4], although the circulatory concentration of cortisol is 200 to 1000-fold higher than that of aldosterone. This paradox was solved with the discovery of the enzyme 11β-hydroxysteroid dehydrogenase type 2 (11β-HSD2), which protects the MR by degrading biologically active cortisol to inactive cortisone [3], [4]. Liquorice contains glycyrrhizinic acid, which in the bowel is metabolized to glycyrrhetinic acid [5], which in turn inhibits 11β-HSD2 and enhances the action of cortisol on the MR [6]. Therefore, the liquorice-induced elevation of blood pressure is mediated via the MR [7].

In the kidneys the MR is also protected from cortisol by 11β-HSD2 [3], [4]. Excessive renal MR activation by cortisol promotes reabsorption of sodium from renal distal tubule and increases excretion of potassium and hydrogen into the urine. This leads to sodium retention, increased extracellular volume, hypertension, hypokalemia and metabolic alkalosis. This condition is called pseudohyperaldosteronism due to low renin and low aldosterone concentrations in plasma, as described in 1968 by Conn [8].

The enzyme 11β-HSD2 is also present in the human vascular wall in both smooth muscle cells and endothelial cells, where it can influence vascular tone by modulating the access of glucocorticoids to the MR [9]. Interestingly, MR blockade can have blood pressure -independent beneficial effects on arterial stiffness [10]. In the heart the presence of 11β-HSD2 is somewhat controversial [1], but it has also been suggested that this enzyme is co-expressed with the MR in the human cardiac tissue [11].

The sensitivity of human subjects to the liquorice-induced elevation of blood pressure varies individually and patients with hypertension show higher sensitivity [12]. Based on the mechanisms described above, the elevation of blood pressure could result from increased extracellular volume caused by sodium retention, enhanced peripheral arterial resistance due to vasoconstriction, increased large arterial stiffness via MR activation, or changes in cardiac function. Since the detailed hemodynamic effects remain unknown, we examined the haemodynamic changes induced by liquorice ingestion in healthy volunteers.

## Materials and Methods

The original study protocol (in Finnish) and English summary of study protocol, and supporting Consort checklist, and data are available as supporting information; see Protocol S1, Protocol S2, Checklist S1 and Data S1.

### Ethics statement

All participants signed informed study consent. This study was approved by the Ethics Committee of the Tampere University Hospital (study code R07053M), complies with the principles outlined in the Declaration of Helsinki, and is registered in the database of clinical trials (ClinicalTrials.gov, ID: NCT01742702).

The haemodynamic recording protocol was registered in the EU Clinical Trials Register (EudraCT-number 2006-002065-39), and the study was approved by the Ethics Committee of Tampere University Hospital and by the Finnish Medicines Agency (code numbers R06086M for controls and R07053M for liquorice diet). The ongoing haemodynamic study was also registered in the database of clinical trials (ClinicalTrials.gov, ID: NCT01742702). The authors confirm that all ongoing and related trials for this intervention are registered.

### Study design and participants

**Figure 1:**
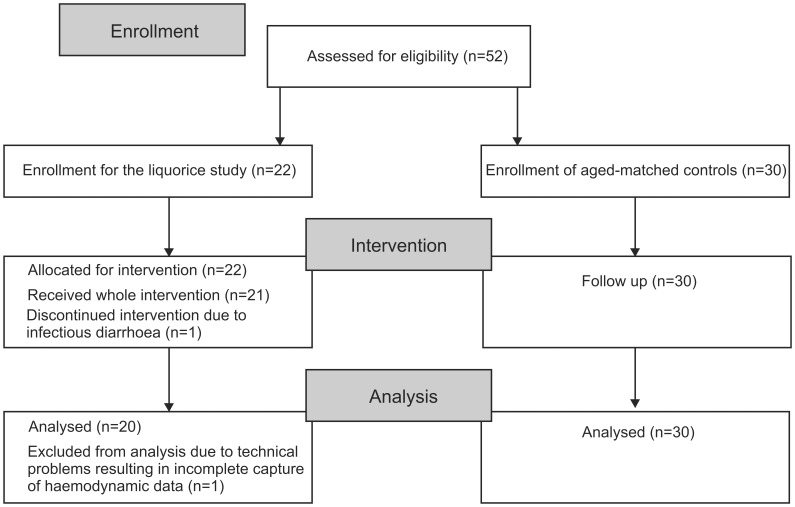
Flow diagram of the study.

This was as an open-label study, and all participants were recorded before and after the 2-week intervention (liquorice ingestion) or the 1–3 week follow-up (normal diet). Experts from a Finnish sweet manufacturer (www.fazer.com) advised us that a product that would have authentic liquorice taste in the absence of glycyrrhizin could not be prepared, and therefore a double-blind crossover study design was not possible. The study subjects were recruited through noticeboard or email announcements from the personnel and students of the University of Tampere and Tampere University Hospital. In addition, professionals in local occupational health care units were informed that their patients could participate in an ongoing study on haemodynamics that included medical inspection and routine laboratory analyses (Figure 1). The data were collected from June 2006 to June 2011 at the Department of Clinical Physiology, Tampere University Hospital, Finland.

The *liquorice group* initially consisted of 22 normotensive volunteers, but one subject discontinued the study due to infectious diarrhoea. In addition, capture of haemodynamic data was incomplete in one subject, who was excluded from final analyses. The final *liquorice group* consisted of 20 subjects (12 women and 8 men, aged 33.6±2 years), who ingested liquorice daily for two weeks. Preceding the study, the subjects were instructed to abstain from liquorice-containing products for 3 weeks. The participants were allowed to choose between two commercial products (Halva liquorice™, Kouvola liquorice™) so that the estimated daily dose of glycyrrhizin was 290–370 mg. If the daily glycyrrhizin dose exceeds 400 mg, the risk of adverse events would be increased [13]. The glycyrrhizin content in these products was determined in 1993 [14], and according to the manufacturers, the preparation processes have not changed. Ethical approval allowed a liquorice intervention for a maximum period of 4 weeks. However, to ensure compliance the study was carried out using a 2-week-long liquorice diet. The dose and duration of the liquorice diet were based on experience gained by two members of the research group, who committed themselves to daily liquorice ingestion.

#### Normal diet group

To distinguish the possible effect of repeated haemodynamic measurements from the cardiovascular effects of liquorice consumption we recruited an age-matched group of 30 people. These subjects were not aware of serving as a control group for the liquorice study and during the measurements they were advised to maintain their normal diet. None in the normal diet group reported to consume liquorice containing products daily or weekly.

Exclusion criteria were office blood pressure over 140/90 mmHg, any cardiovascular disease with regular medication, or pregnancy. The participants were advised to inform the study group immediately if they gained weight over 4 kilograms, observed oedema in the lower extremities or encountered other problems during liquorice ingestion. Such problems were not reported, and 21 subjects maintained the liquorice diet for 2 weeks.

The suitability of all subjects for the study was confirmed by medical and laboratory examinations. The lifestyle habits, medical history and family history were recorded. In the liquorice group one subject was on regular medication for asthma (160 µg budesonide +4.5 µg formoterol twice daily), one for depression (escitalopram 5 mg once daily), one on postmenopausal oestrogen replacement therapy, and five female subjects used oral contraceptives. In the normal diet group 5 female subjects used oral contraceptives and 3 had hormonal intrauterine devices. There were 4 current smokers in the liquorice diet group, and 3 current and 4 previous smokers in the normal diet group.

### Laboratory analyses

Standard electrocardiograms with 12 leads were taken with MAC5000 (GE Healthcare, Chalfont St. Giles, UK), and the recordings were normal in all participants. Before the study, blood and urine samples were collected after 12 hours of fasting, while in the normal diet group laboratory samples were not obtained from one subject. Plasma sodium, potassium, creatinine, glucose, cholesterol lipoproteins were analysed using Cobas Integra 700/800 (F. Hoffmann-LaRoche Ltd, Basel, Switzerland), and blood haemoglobin by ADVIA 120 or 2120 (Bayer Health Care, Tarrytown, NY, USA). Plasma aldosterone concentration was determined using radioimmunoassay (Aldosterone RIA Test DSL-8600, Diagnostics Systems Laboratories Inc, Webster, TX, USA).

### Blood pressure monitoring and pulse wave analysis

Blood pressure and pulse wave form were continuously recorded for 5 minutes from the left radial artery by a tonometric sensor (Colin BP-508T, Colin Medical Instruments Corp., USA). The tonometric recordings were calibrated twice during every 5-minute period using automated contralateral brachial blood pressure measurements. The SphygmoCor PWMx pulse wave monitoring system (Atcor Medical, Australia) was used to derive aortic blood pressure by previously reported generalized transfer function [15]. Heart rate, aortic pulse pressure (PP), augmentation index (AIx) (augmentation pressure/PP*100), and AIx adjusted to heart rate 75/min (AIx@75) were also determined. In addition, blood pressure was monitored from the left 3rd finger (Finometer^R^ model 1, Finapres Medical Systems, Amsterdam, the Netherlands).

### Whole-body impedance cardiography

Whole-body impedance cardiography (CircMon^R^, JR Medical Ltd., Tallinn, Estonia), based on the changes in electrical impedance of the body during cardiac cycle, was used to determine beat-to beat heart rate, stroke volume, cardiac index, extracellular volume, and pulse wave velocity (PWV) [16]. Systemic vascular resistance index was calculated from radial blood pressure and cardiac index [17]. With the Circmon^R^ whole-body impedance cardiography method, i) the measurement of stroke volume shows good correlation with 3-dimensional echocardiography recordings [18], ii) the measurement of cardiac output shows good agreement with the thermodilution method [16], and the reproducibility and repeatability of both cardiac output and PWV measurements is good [16], [18].

### Haemodynamic measurements

Before recordings of pulse wave analysis and whole-body impedance cardiography, brachial systolic and diastolic blood pressure (SBP and DBP, respectively) was manually measured in the supine position using a standard sphygmomanometer, and Korotkoff sounds at phase V were used for estimating DBP.

The haemodynamic measurements were performed in a temperature-controlled laboratory before and after 2 weeks of liquorice ingestion. In the normal diet group the interval between the recordings was 1–3 weeks. The subjects were in the supine position on the examination table with electrodes on the surface of thorax and distally on the medial surface of extremities, tonometric sensor for pulse wave analysis on the left radial artery, and a cuff for the calibration of radial blood pressure in the right brachium [17]. Plethysmographic blood pressure sensor was placed on the 3^rd^ left finger. The extended left upper limb was held at the level of the heart. Hemodynamic variables were captured continuously for 5 minutes, and average values from the whole period were used in the analyses. We have previously shown the good repeatability and reproducibility of the measurements [19].

### Statistical analyses

The primary endpoints were significant changes in BP and other haemodynamic variables during the study. Secondary endpoints were changes in weight and plasma aldosterone and potassium concentrations. The power calculations were based on expected changes in SBP according to the primary aim of the study. The required minimum sample size was 17 experimental and 26 control subjects to detect a 9 mmHg difference in the change in SBP from baseline (alpha level 0.05, 80% power, standard deviation (SD) 10 for variability, power analysis method for two-sample t-test). Normally distributed data was expressed as mean ± SD or 95% confidence interval (95% CI). The results are given as mean ± SD unless otherwise stated. General linear model of repeated measurements and t-test for paired samples were used to compare differences between measurements and groups. A chi-square test was used to compare gender distribution in groups, and Pearson's or Spearman's correlations (r) were calculated, as appropriate. *P*<0.05 was considered significant. Statistical analyses were made using SPSS 17.0 (SPSS Inc., Chicago, Ill., USA).

## Results

Demographic data and baseline laboratory values are presented in Table 1. At baseline there were no significant differences in any of the measured variables between the groups. Extracellular water and weight did not change during the normal diet, but after two weeks of liquorice diet extracellular water volume increased by 0.5 (±1.4) litres and body weight by 1.5 kg (±1.5) (*P* = 0.04 and P = 0.002, between the groups, respectively) (Table 2).

**Table 1:** Demographic data and laboratory values at baseline.

	Liquorice diet n = 20	Normal diet n = 29–30*	*P* value
Sex (F/M)	12/8	17/13	0.53
Age (years)	33.5±7.9	33.8±8.1	0.91
Height (cm)	174±9	174±8	0.82
Body mass index (kg/m^2^)	23.3±1.9	23.0±2.7	0.69
Haemoglobin (g/l)	140±8	143±15	0.40
Fasting plasma			
Cholesterol (mmol/l)	4.5±0.7	4.4±0.8	0.48
Triglycerides (mmol/l)	0.8±0.3	0.9±0.5	0.36
High density lipoprotein (mmol/l)	1.8±0.4	1.7±0.4	0.37
Low density lipoprotein (mmol/l)	2.3±0.6	2.2±0.7	0.59
Glucose (mmol/l)	5.2±0.4	5.0±0.4	0.13
Creatinine (µmol/l)	79±15	76±11	0.39
Sodium (mmol/l)	140±1.2	140±2.0	0.84
Potassium (mmol/l)	3.9±0.3	3.9±0.2	0.73

All other values are mean ± SD except for sex, which shows the number of subjects.

*Blood samples for fasting plasma values were not obtained from one subject.

**Table 2:** Changes in weight, extracellular water, brachial office systolic and diastolic blood pressure, plasma aldosterone and plasma potassium after two weeks of liquorice ingestion.

	Liquorice diet n = 20	Normal diet n = 29–30*	*P* value
Weight (kg)			
Before liquorice ingestion	70±9	71±9	0.73
After liquorice ingestion	72±9^‡^	71±8	0.87
Extracellular water (l)			
Before liquorice ingestion	12.5±1.4	12.7±1.2	0.47
Change after liquorice ingestion	0.5±1.4	−0.1±0.5	0.04
Office systolic blood pressure (mmHg)			
Before liquorice ingestion	120±8	116±14	0.24
Change after liquorice ingestion	1.7±7.0	−3.1±4.9	0.01
Office diastolic blood pressure (mmHg)			
Before liquorice ingestion	71±8	72±11	0.76
Change after liquorice ingestion	2.8±5.0	−3.5±6.2	0.001
Aldosterone (pmol/l)			
Before liquorice ingestion	522±277	840±1202#	0.25
After liquorice ingestion	197±75^‡^	not determined	-
Potassium (mmol/l)			
Before liquorice ingestion	3.9±0.3	3.9±0.2	0.73
After liquorice ingestion	3.7±0.3†	not determined	-

All values are mean ± SD.

*Blood samples for fasting plasma values were not obtained from one subject.

† *P* = 0.02 and ^‡^ *P*<0.001 versus the corresponding value before liquorice diet.

# Two normotensive and normokalemic subjects showed divergent serum aldosterone concentrations (6138 and 3720 pmol/l). If these subjects were excluded from the analysis, mean serum aldosterone concentration in the normal diet group was 548±340 pmol/l (p = 0.78 between groups).

In manual measurements, brachial SBP/DBP increased by 2/3 (±7/5) mmHg in the liquorice diet group (*P* = 0.310 for SBP and *P* = 0.031 for DBP) and decreased by −3/−4 (±5/6) mmHg in the normal diet group (*P* = 0.002 for SBP and *P* = 0.004 for DBP) (Table 2) during the study. These moderate changes in SBP and DBP were significantly different between the groups (*P* = 0.007 for SBP and *P* = 0.001 for DBP). Liquorice ingestion significantly decreased plasma aldosterone (*P*<0.001) and potassium concentrations (*P* = 0.02) (Table 2).

**Figure 2:**
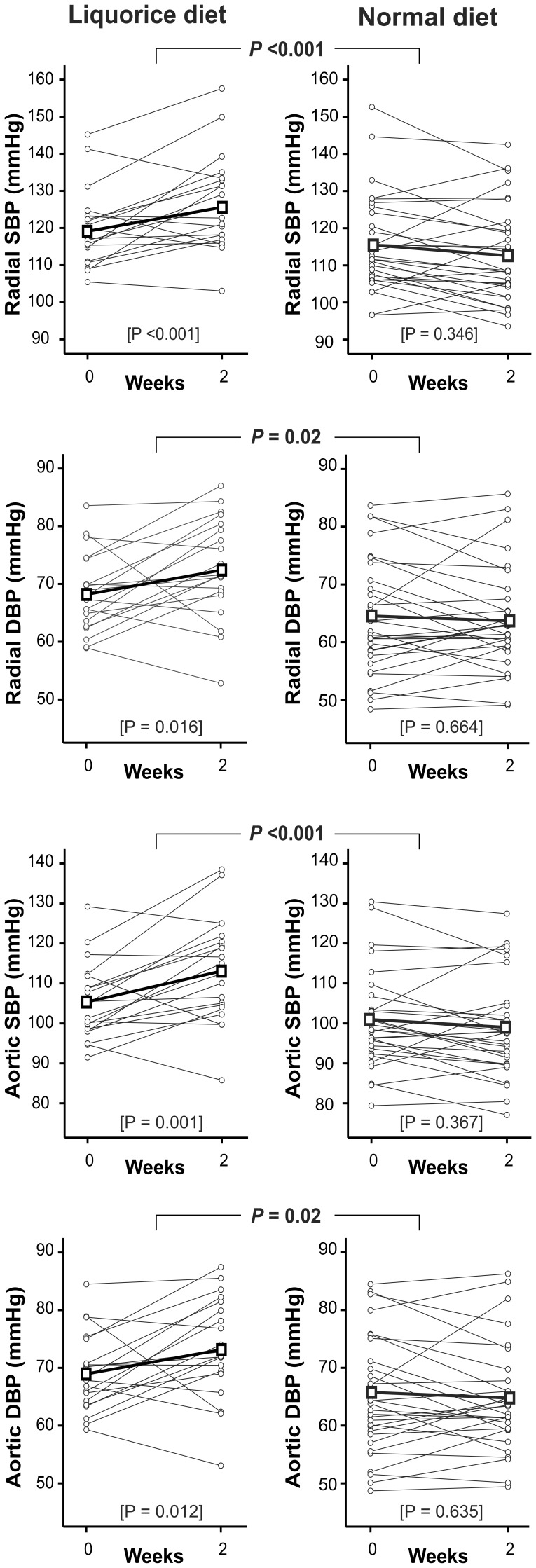
Radial and aortic blood pressure, liquorice diet versus normal diet. Grey lines represent each individual, thick black line represents mean values of each variable. SBP, systolic blood pressure; DBP, diastolic blood pressure; *P* values between graphs are for the difference in the change of each variable between liquorice diet versus normal diet, *P* values in brackets are for the change within each group, n = 20 and 30 in the liquorice and normal diet groups, respectively.

In tonometric measurements, the mean change (95% CI) between the visits in radial SBP/DBP in the liquorice group was 7/4 (2-11/1-8) mmHg (within group *P*-value <0.001 for SBP and *P* = 0.016 for DBP) and in the normal diet group −3/−0.3 (−5-0.4/−3-2) mmHg (*P* = 0.346 for SBP and *P* = 0.664 for DBP). The differences between the groups were significant (*P*<0.001 for radial SBP and *P* = 0.02 for radial DBP) (Figure 2). The manual measurements of brachial SBP and DBP correlated well with tonometric radial measurements (during visit 2, r = 0.85 for SBP, P<0.001; r = 0.81 for DBP, P<0.001).

Comparable changes in blood pressure were also observed in finger measurements, as the mean change (95% CI) between the visits in finger SBP/DBP in the liquorice group was 5/4 (1-9/−1-8) mmHg (within group *P* = 0.010 for SBP and *P* = 0.102 for DBP), and in the normal diet group −2/−2 (−7-3/−5-2) mmHg (*P* = 0.402 for SBP and *P* = 0.352 for DBP). The mean change in finger SBP differed between the groups (*P* = 0.030), while the change in DBP did not reach statistical significance (*P* = 0.065). The manual measurements of brachial blood pressure and the plethysmographic measurements of finger blood pressure confirmed the significant effect of the liquorice intervention on blood pressure.

Aortic SBP/DBP increased in the liquorice group (8/4 mmHg, 95% CI 3-13/1-8, *P* = 0.001 for SBP and *P* = 0.012 for DBP), while no significant change was observed in the normal diet group (−2/−0.5 mmHg, 95% CI −4-1/−3-2, *P* = 0.367 for SBP and *P* = 0.635 for DBP). The mean change between the visits in aortic SBP/DBP differed between the groups (*P*<0.001 for aortic SBP and *P* = 0.02 for aortic DBP, Figure 2). No correlations were found between the change in weight and the changes in radial or aortic blood pressures, and the change in extracellular water did not correlate with the changes in radial or aortic blood pressures, either (data not shown).

**Figure 3:**
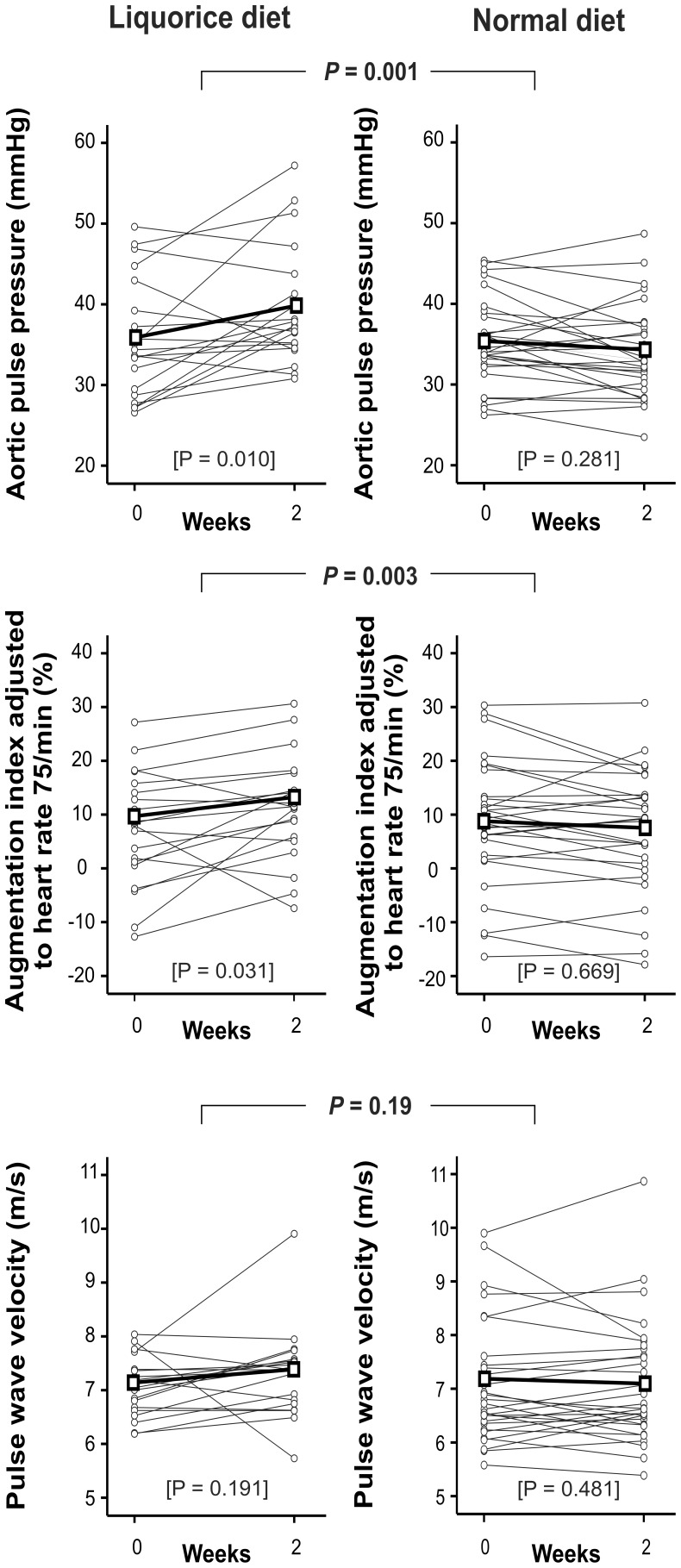
Aortic pulse pressure, augmentation index, and pulse wave velocity, liquorice diet versus normal diet. Grey lines represent each individual, thick black line represents mean values of each variable. *P* values are for the difference in the change of each variable between liquorice diet versus normal diet, *P* values in brackets are for the change within each group, n = 20 and 30 in the liquorice and normal diet groups, respectively.

In the liquorice group aortic PP, AIx, and AIx@75 increased significantly: aortic PP was elevated by 4 (1-7) mmHg (*P* = 0.01, Figure 3), AIx increased from 13.7% to 18.7% (95% CI for change 1.3-8.9, P = 0.012), and AIx@75 increased from 7.3% to 11.2% (95% CI for change 0.3-7.5, *P* = 0.031, Figure 3). In the normal diet group aortic PP, AIx and AIx@75 did not significantly change between the visits: the difference in aortic PP was −1 (95% CI −3-0.2) mmHg (*P* = 0.281), AIx values were 13.3% vs. 12.4% (95% CI for change −2.9-1.1, *P* = 0.355), and AIx@75 values were 8.6% vs. 6.9% (95% CI for change −3.4-0.2, *P* = 0.669). The differences between the groups were significant for aortic PP, AIx, and AIx@75 (*P* = 0.001, *P* = 0.003, and *P* = 0.003, respectively).

**Figure 4:**
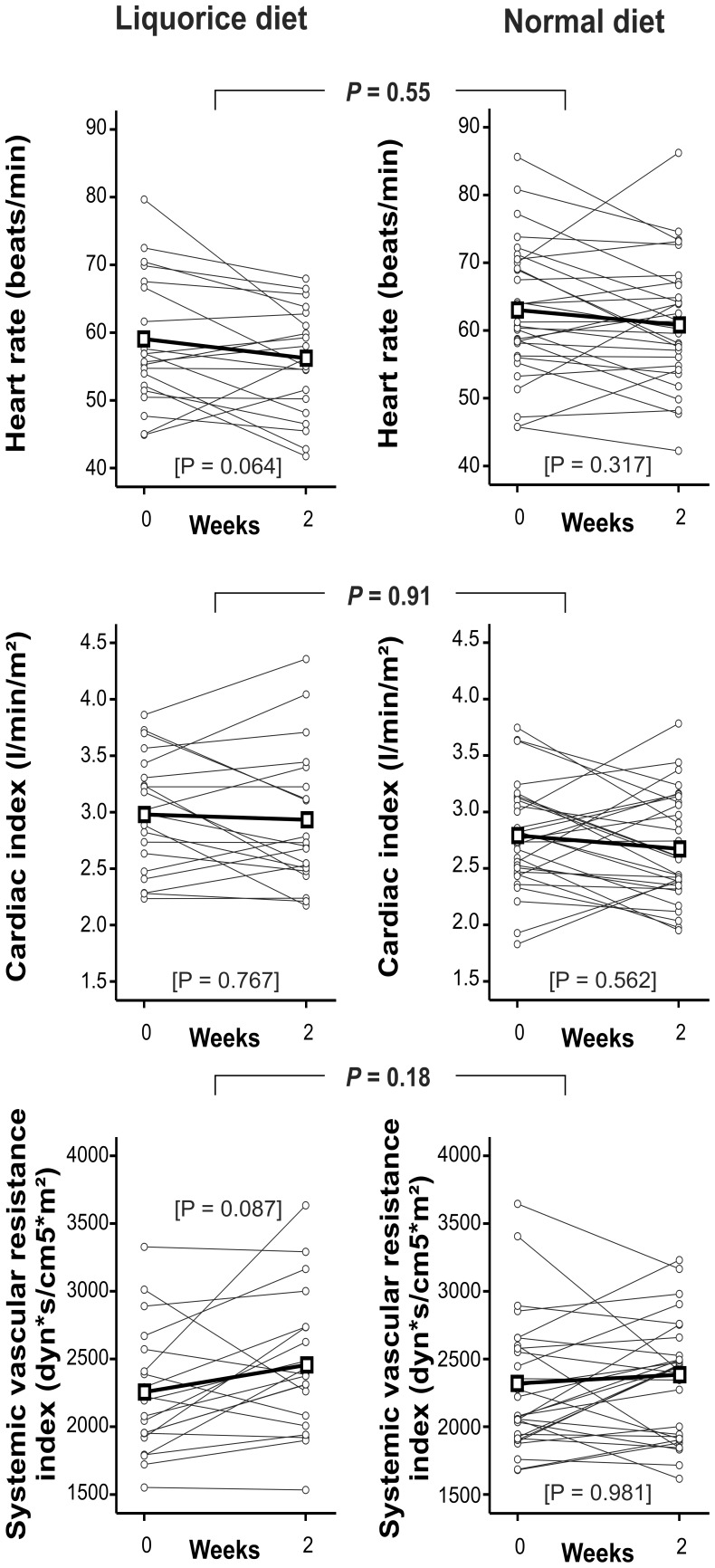
Heart rate, cardiac index, systemic vascular resistance index, liquorice diet versus normal diet. Grey lines represent each individual, thick black line represents mean change of each variable. *P* values are for the difference in the change of each variable between liquorice diet versus normal diet, *P* values in brackets are for the change within each group, n = 20 and 30 in the liquorice and normal diet groups, respectively.

PWV, an acknowledged marker of arterial stiffness, did not significantly change in either group (*P* = 0.191 and *P* = 0.481, liquorice and normal diet groups, respectively), and did not significantly differ between the groups (*P* = 0.19, Figure 3). The small changes in PWV, albeit statistically insignificant, correlated with changes in radial and aortic SBP and DBP (r = 0.40–0.45 for all, *P* values ranging 0.001–0.005). There were no significant differences in heart rate, cardiac index, and systemic vascular resistance index in the measurements within the groups (*P*>0.05 for all), or between the groups (*P* = 0.55, 0.91 and 0.18 for heart rate, cardiac index and systemic vascular resistance index, respectively, Figure 4).

## Discussion

Although liquorice ingestion is a known cause for diet-induced hypertension, the detailed haemodynamic changes underlying the elevation of blood pressure have not been reported after regular liquorice exposure. In this study we captured haemodynamic data before and after two weeks of liquorice ingestion, and found that the diet elevated both peripheral and central blood pressure and increased extracellular volume. The findings that central PP, AIx, and AIx@75 were also increased following the liquorice diet indicate that pressure wave reflection from the peripheral circulation was enhanced. In contrast, PWV, an acknowledged marker of arterial stiffness, did not significantly change, and neither did cardiac output nor systemic vascular resistance. Although blood pressure has been defined as the product (not sum) of cardiac output and peripheral vascular resistance [20], moderate changes in the level of blood pressure are thus possible in the absence of statistically significant changes in these two principal variables. Taken together, the liquorice-induced elevation of blood pressure was associated with increased extracellular volume and enhanced pressure wave reflection from the peripheral arterial tree.

The recordings of tonometric radial and plethysmographic finger blood pressure, pulse wave analysis, and whole-body impedance cardiography were automated procedures devoid of subjective components, which can be argued to increase the reliability of the recordings. The study groups were homogenous so that different haemodynamic profiles associated e.g. with excess body weight and ageing could be avoided [21]. Although individual differences exist in the metabolism of glycyrrhizin to glycyrrhetinic acid in the bowel and in the absorption of the latter, the glycyrrhizin content (w/w%) of the consumed product is the most important determinant of the clinical response to liquorice [22]. In the present study, increased extracellular volume and decreased plasma aldosterone and potassium levels documented the compliance of the participants as obvious consequences of the MR activation. Of note, subjects in the normal diet group were unaware that they served as controls for the liquorice diet, so they could not subconsciously change their dietary habits regarding liquorice containing products.

The type 1 and type 2 isoforms of 11β-HSDs regulate local cortisol metabolism. The 11β-HSD2 is mainly expressed in aldosterone target tissues [23], while both 11β-HSD isoenzymes are present in the vascular wall [24]. Arteries are thus a target tissue for mineralocorticoids and glucocorticoids, which via the MR and GR can potentiate vascular responses to pressor hormones like α-adrenoceptor agonists and angiotensin II [25]. Of note, the effects of liquorice ingestion on 11β-HSD2, plasma electrolytes, and the renin-angiotensin-aldosterone axis can be long-lasting, since abnormalities in plasma electrolyte levels and urinary cortisol excretion may persist for 1–2 weeks after cessation of liquorice ingestion [26]. Moreover, after prolonged regular liquorice ingestion, the normalization of suppressed plasma renin and aldosterone values may take up to 2–4 months [27].

Recent studies have suggested that central blood pressure and PP are superior to brachial blood pressure in evaluating the risk of future cardiovascular events [28]–[30]. In a meta-analysis the predictive value of central AIx on cardiovascular events and all-cause mortality was even suggested to be independent of the level of blood pressure and heart rate [31]. In the present study, AIx was increased after liquorice diet, but PWV, an acknowledged measure of arterial stiffness, was not changed. Wave reflection occurs predominantly at the origin of the terminations of low resistance arteries into high-resistance arterioles, and stiffening of large arteries is known to result in earlier return of the reflected wave and higher AIx [32]. When large arterial stiffening results in earlier return of the reflected waves from the periphery to the heart, this process increases aortic SBP but reduces aortic DBP [32], [33]. Thus, the present finding of increased aortic DBP following the liquorice diet agrees with the view that changes in arterial stiffness were not the cause for the alterations in central BP. Importantly, the level of AIx is not only determined by arterial stiffness, but also by systemic vascular resistance and other factors including sex, height and heart rate; actually the detailed determinants of AIx are still a matter of debate [32]–[34]. It seems possible that increased extracellular volume might also have an influence on the magnitude of AIx. Finally, it should be noted that arterial stiffness depends greatly on the prevailing blood pressure that is distending the blood vessels [35]. Although the present small changes in PWV were not statistically significant, they correlated with parallel changes in radial and aortic SBP and DBP. The present 2-week observation period was too short for structural changes to take place that would increase arterial stiffness, but such changes can be anticipated to result from longer periods of elevated blood pressure [36].

In the present study, the elevation of SBP and DBP caused by liquorice (on average 7/4 mmHg) was close to the efficacy of antihypertensive monotherapy in meta-analysis on pharmacotherapy of hypertension (average reduction 9/6 mmHg) [37]. Liquorice ingestion should be considered when taking the history of a hypertensive patient, but subjects may not always be aware that they have consumed liquorice containing products. In addition to sweets, many other food supplies contain glycyrrhizin as a sweetener or flavouring agent, including confectionery, beverages, alcohol drinks, cough mixtures, herbal medicines, health foods, and chewing tobacco. Moreover, dozens of medicinal preparations contain liquorice or liquorice derivatives [38]. According to the Finnish customs (www.tulli.fi), the amount of imported liquorice root extract was 686.000 kg during a period of 12 months (statistics from May 2013), and this would correspond to a daily dose of glycyrrhizin of approximately 21 mg per every Finnish citizen. Although glycyrrhizin containing products are exported, this indicates high domestic consumption of liquorice products.

A limitation of the present study is the rather small number of participants, although significant haemodynamic changes were observed in the 20 subjects. Due to the small number of subjects, the possible blood pressure-independent haemodynamic effects of liquorice could not be examined by the use of multivariate analysis. However, as the effect of liquorice on blood pressure is well-established, the objective of the present study was to examine the haemodynamic changes that can explain the elevation of blood pressure. Furthermore, in addition to glycyrrhizin, the present liquorice diet also contained carbohydrates, mainly starch-derived glucose. The average intake of carbohydrates from liquorice was 150 grams per day, which could have influenced cardiovascular status. Diets with high glycaemic index are also associated with higher degree of vascular oxidative stress [39]. However, liquorice root extract contains many natural antioxidants [40], and the balance between these two factors remains unknown. The rapid 2 kg weight gain during the study was most likely due to fluid retention, since the total intake of carbohydrates from liquorice was about 2 kg during the 2-week period. The bioimpedance measurements suggested that extracellular water was increased by approximately 0.5 kg, but possible changes in the volume of intracellular water remain unknown. The observed change in weight did not correlate with changes in blood pressure, in parallel with previous findings by Sigurjonsdottir et al. showing a corresponding increase in weight following liquorice ingestion that was not associated with changes in blood pressure [12].

In order to avoid the excess carbohydrate intake, the participants could be given pure glycyrrhizin. However, we especially tested the hypothesis whether daily glycyrrhizin intake from liquorice would induce changes in cardiovascular regulation that could be detected with our recording system. As the effect of liquorice may differ between sexes, the present study subjects were chosen so that the distribution of genders in the groups did not differ. In a study concerning individual sensitivity to the harmful influences of liquorice intake, women were suggested to be more sensitive to the adverse effects of liquorice than men [12]. In contrast, a later study by the same group showed that plasma aldosterone levels were lower in men than in women during 11β-HSD2 inhibition with liquorice [41].

In conclusion, the results of this study with normal volunteers indicated that already two weeks of liquorice ingestion increased extracellular volume, amplified pressure wave reflection from the peripheral circulation, and elevated central systolic and diastolic blood pressure.

## Supporting Information

Protocol S1 **Trial protocol.**
(PDF)

Protocol S2 **English summary of trial protocol.**
(PDF)

Checklist S1 **CONSORT checklist.**
(PDF)

Data S1 **Leskinen data.**
(TXT)
