# Supplementary material for: Daily Liquorice Consumption for Two Weeks Increases Augmentation Index and Central Systolic and Diastolic Blood Pressure
Source: PLoS One. 2014 Aug 25;9(8):e105607. doi: 10.1371/journal.pone.0105607 (PMC4143270; doi:10.1371/journal.pone.0105607)
Supplement: Protocol S1 — Trial protocol. (PDF) [file pone.0105607.s001.pdf]

PROTOCOL S1

Lakritsin vaikutus hemodynamiikkaan - DYNAMIC-tutkimuksen osahanke

Miia Leskinen, LK (LL 4/2007), tutkija, TaY
Ilkka Pörsti, professori, oyl, TaY/TAYS, sisätaudit
Anna Tahvanainen, LL, tutkija, TaY
Jenni Koskela, LL, tutkija, TaY
Mika Kähönen, professori, oyl, TaY/TAYS, kliininen fysiologia
Terho Lehtimäki, professori, yl, TaY/TAYS, kliininen kemia
Tiit Kööbi, dosentti, yl, TAYS, kliininen fysiologia
Jukka Mustonen, professori, yl, TaY/TAYS, sisätaudit

Vastaava tutkija
Prof. Ilkka Pörsti
TaY Lääketieteen laitos / sisätaudit
33014 Tampereen yliopisto

1. Tutkimussuunnitelman yhteenveto

Kohonneeseen verenpaineeseen liittyy muutoksia verisuonten rakenteessa ja toiminnassa (sileälihas, endoteeli) sekä lisääntynyt ääreisverenkierron vastus, mitkä ovat nykymenetelmin todettavissa jo ennen epäsuorasti mitatun verenpaineen nousua. Runsaan lakritsin syönnin aiheuttama hypertensio on todennäköisesti ajateltua yleisempää [1, 2], ja osa väestöstä on erityisen herkkää lakritsin verenpainetta nostavalle vaikutukselle [3]. Lakritsihypertension tunnistaminen on mahdollista siten, että mitataan plasman reniiniaktiivisuus sekä määritetään plasman ja virtsan aldosteronipitoisuudet tai kortisoli ja sen metaboliitit vuorokausivirtsasta. Lakritsihypertensiossa reniini ja aldosteroni ovat matalat, kortisolin katabolia estyy, eikä potilailla ole yleensä todettavissa hypokalemiaa. On todennäköistä, että lakritsin aiheuttamat hemodynaamiset muutokset ovat mitattavissa ennen verenpaineen nousua.

Tämä tutkimus liittyy DYNAMIC-tutkimukseen, jolle on 5/2006 myönnetty Lääkelaitoksen ja PSHP:n kuntayhtymän Eettisen toimikunnan lupa (R06086M). Tutkimuksessa suoritetaan DYNAMIC-tutkimuksen protokollan mukaan enintään 40 koehenkilöille kajoamattomat hemodynaamiset mittaukset levossa ja 5 minuutin mittaisen toistetun kallistuskokeen aikana impedanssikardiografiaa, pletysmografista sormipaineiden mittausta ja jatkuvaa tonometrista pulssiaallon analyysijärjestelmää käyttäen. Mittausten avulla saadaan käsitys nestetasapainosta, endoteelin toiminnasta, verisuonten laajenemisesta ja supistumistaipumuksesta, verisuonten myötäävyydestä, baroreseptorijärjestelmästä ja autonomisen hermoston toiminnasta. Tutkimuslääkkeinä mittausten yhteydessä käytetään 0.25 mg sublinguaalista nitroglyseriiniä ja 400 µg inhaloitavaa salbutamolia.

Ensimmäisten hemodynaamisten mittauksien jälkeen vapaaehtoiset koehenkilöt nauttivat 60-300 g lakritsia päivässä valmisteen glykyrritsiinipitoisuudesta riippuen korkeintaan 4 viikon ajan (päivittäinen glykyrritsiiniannos enintään 400 mg). Uudet hemodynaamiset mittaukset ja hormonaaliset määritykset tehdään ensimmäisen lakritsialtistusviikon jälkeen sekä altistuksen lopussa. Vertaamalla jälkimmäisiä hemodynaamisia mittauksia ennen lakritsialtistuksen aloitusta tehtyyn mittaukseen voidaan muodostaa käsitys lakritsin syönnin vaikutuksesta verenkierron säätelyyn.

2.Taustaa

Lakritsi on suomalaisille tuttu aine lähinnä makeisista, joissa makuaineena käytetään pieniä määriä lakritsikasvin juurijauhetta tai –uutetta. Elintarvikkeiden lisäksi lakritsin juuriuutetta on joissakin yskänlääkkeissä ja luontaistuotteissa [4]. Lakritsilla on jo pitkään tiedetty olevan fysiologisia vaikutuksia, joista tunnetuin lienee sen taipumus kohottaa verenpainetta.

2.1 Glykyrritsiini ja sen vaikutusmekanismi

Lakritsikasvin (Glycyrrhiza glabra) juuri sisältää lukuisia aineita, joista farmakologisesti tärkein ja tutkituin on glykyrritsiini eli glykyrritsiinihappo. Glykyrritsiini metaboloituu suolistossa glykyrretiinihapoksi, jonka imeytyminen on lähes täydellistä. Glykyrretiinihappo muistuttaa rakenteeltaan steroideja ja se pystyy vaikuttamaan glukokortikoidimetaboliaan estämällä 11-β-hydroksisteroididehydrogenaasi-tyyppi-2:n (11-β-OHSD2) toimintaa. Tämä entsyymi muuttaa biologisesti aktiivisen kortisolin inaktiiviseksi kortisoniksi mm. munuaisen tubulussoluissa ja sen toimimattomuus aiheuttaa munuaiskudoksessa solunsisäisen kortisolipitoisuuden kasvun [5-7]. Tämä on merkittävää, koska glukokortikoidireseptorien lisäksi kortisoli pystyy sitoutumaan tehokkaasti munuaisissa ja muissa kudoksissa myös mineralokortikoidireseptoreihin (aldosteronireseptori) [8]. Kortisolipitoisuuden kasvusta munuaiskudoksessa seuraakin hyperaldosteronismin kaltainen tila, jossa aldosteronireseptorin aktivoituminen tehostaa natriumin takaisinottoa ja kaliumia ja vetyioneja menetetään enemmän virtsaan. Seurauksena on elimistön natriumin määrän lisääntyminen ja solunulkoisen nestetilavuuden kasvu, hypokalemia sekä metabolinen alkaloosi. Reniini- ja aldosteronitasot ovat matalat, päinvastoin kuin hyperaldosteronismissa. Tilaa voidaan kutsua pseudohyperaldosteronismiksi [9].

2.2 Lakritsioireyhtymä

Liiallinen lakritsivalmisteiden nauttiminen voi aiheuttaa lakritsioireyhtymänä tunnetun tilan [1, 2]. Tilaa pidetään alidiagnosoituna [2]. Sen löydöksiä ovat kohonnut verenpaine, turvotukset ja matalat plasman reniini- ja aldosteronitasot, mutta vain osalla potilaista on hypokalemia [10]. Lakritsioireyhtymä on merkittävä sekundaarisen kohonneen verenpaineen aiheuttaja, ja se johtuu glykyrritsiinin vaikutuksesta glukokortikoidimetaboliaan. Tätä vaikutusta voidaan mitata biokemiallisesti määrittämällä vuorokausivirtsasta kortisoli, kortisoni sekä näiden tetrahydrometaboliittien (tetrahydrokortisoli, alloterahydrokortisoli, tetrahydrokortisoni) pitoisuudet. 11-β-OHSD2:n toiminnan estyessä näiden pitoisuudet virtsassa kasvavat [5, 6].

Tutkimusten mukaan ihmisten herkkyys lakritsin haittavaikutuksille vaihtelee yksilöllisesti, ja esimerkiksi essentielliä verenpainetautia sairastavat ovat terveitä herkempiä lakritsin vaikutuksille [3]. Myös nautitun lakritsin glykyrritsiinipitoisuudella sekä lakritsin säännöllisen käytön pituudella on merkitystä haittavaikutusten synnyssä. Elintarvikevirasto tutki 1990-luvun alussa, että Suomessa myytävissä lakritsimakeisissa oli keskimäärin 0,2% glykyrritsiiniä, mutta glykyrritsiinipitoisuuksien vaihteluväli tässä tutkimuksessa oli laaja: 0,017-0,73% [11]. Erityisen herkät henkilöt voivat saada haittavaikutuksia jo 100 mg:n päivittäisestä glykyrritsiinin saannista, mutta vasta 400 mg:n ylittävä säännöllinen päiväannos glykyrritsiiniä aiheuttaa haittoja useimmille [12]. Yleisimmät haittavaikutukset ovat muutokset elektrolyyttitasapainossa, reniini-angiotensiini-aldosteroni-järjestelmän vaimeneminen, kohtalainen verenpaineen nousu, turvotukset sekä painonnousu. Näiden haittavaikutusten vaikeusaste on suhteessa nautittuun päivittäiseen glykyrritsiinimäärään sekä altistuksen pituuteen [12].

2.3 Kortisolin vaikutus hemodynamiikkaan

Elimistön kohonneen kortisolipitoisuuden yhteyttä kohonneeseen verenpaineeseen on tutkittu paljon Cushingin oireyhtymään liittyen. Glukokortikoidit kohottavat verenpainetta mineralokortikoidiaktiivisuutensa kautta, aktivoimalla reniini-angiotensiini-järjestelmää (RAS), lisäämällä inotrooppisten ja vasoaktiivisten aineiden (katekoliamiinit, vasopressiini, angiotensiini II) sekä vähentämällä vasodilataattorien (mm. typpioksidi) vaikutusta verisuoniin [13-16]. Näiden eri mekanismien yhteisvaikutuksena glukokortikoidit nostavat sydämen minuuttitilavuutta, verenkierron kokonaisääreisvastusta sekä munuaisten verenkierron vastusta ja verenpaine nousee [13].

Eksogeeninen kortisolihoito vaikuttaa myös hemodynamiikkaan lisäten sydämen minuuttitilavuutta noin litralla ja kasvattamalla sydämen iskutilavuutta [17]. Verenpaineen nousu voi kuitenkin olla riippumaton sydämen minuuttitilavuuden kasvusta. Vaikka kaikilla tutkituilla kortisoli ei nostanut laskennallista verenkierron kokonaisääreisvastusta, munuaisten verenkierron vastus kasvoi [17]. Koska lakritsin farmakologisesti vaikuttava aine glykyrritsiini nostaa munuaisten kortisolipitoisuutta, voidaan olettaa että lakritsilla on verenpainetta kohottavan vaikutuksen lisäksi myös muita vaikutuksia verenkierron säätelyyn.

3. Tutkimuksen hypoteesi ja tavoitteet

Glukokortikoidikatabolian estyminen soluissa vaikuttaa elimistössä hyvin laajasti ja lisääntyneen kortisolipitoisuuden on todettu nostavan verenkierron ääreisvastusta [13, 18-22]. Tutkimuksemme päähypoteesina on, että lakritsin säännöllisellä nauttimisella on selvä ja mitattava vaikutus verenkierron säätelyyn edellä mainittujen hormonaalisten muutosten vuoksi jo ennen kuin olkavarresta epäsuorasti mitattu verenpaine todetaan kohonneeksi.

Tämän DYNAMIC-tutkimuksen metodia käyttävän lakritsitutkimuksen tavoitteena on saada lisätietoa säännöllisen lakritsin syönnin vaikutuksista verenkierron säätelyyn. Tarkoitus on selvittää, aiheuttaako lakritsin syönti kaikille tutkittaville mitattavia muutoksia verenkierron säätelyssä ja mitä nämä mahdolliset muuttujat ovat. Lisäksi tavoitteena on selvittää, että voidaanko hemodynaamisten muuttujien avulla tunnistaa lakritsin haitoille poikkeuksellisen herkät yksilöt. Tärkeänä tavoitteena on myös luoda uusia keinoja ja välineitä sekundaarisen hypertensiopotilaan tunnistamiseen, ja vähentää tältä osin näiden potilaiden alidiagnosointia. Lakritsin nauttimisesta aiheutuneen hypertension tunnistaminen on tärkeää, koska tila korjaantuu lakritsin syönnin lopettamisella ja turhat lääkehoidot ovat näin vältettävissä.

4. Menetelmät ja näytteet

4.1 Koko kehon impedanssikardiografia, pulssiaaltoanalyysi ja TILT-koe yhdistettynä lääkealtisteisiin

DYNAMIC-tutkimuksen protokollan mukaan tutkittavilta kerätään terveyttä ja elintapoja kartoittavat tiedot, laboratoriokokeiden ja mittausten tulokset, sekä otetaan verinäytteet peruslaboratoriotutkimuksia ja geneettisiä analyysejä varten. Hemodynamiikan mittaus suoritetaan impedanssikardiografiaa (CircMon), pletysmografista sormipaineiden mittausta (Finapress), tonometrista radialispaineiden mittausta (Colin BP-508T) ja pulssiaallon analyysijärjestelmää (SphygmoCor PVMx) käyt-

täen. Mittaukset suoritetaan sekä makuulla että kallistuskokeen aikana yhdistettynä lääkealtisteisiin (sublinguaalinen nitroglyseriini 0.25 mg ja inhaloitu salbutamoli 400 µg). Mittauskohteita ovat verenpaine, ääreisvastus, sydämen minuuttivolyymi ja pumppufunktio, pulssiaallon etenemisnopeus ja sentraalisten suonten paineolosuhteet. Laitteisto antaa validoitua informaatiota verenkiertoelimistöstä (Food and Drug Administration USA 2002; hyväksyntänumerot: PVx K012487, Mx K002742). Mittaukset suoritetaan TAYS:n kliinisen fysiologian ja isotooppi-lääketieteen yksikössä (Suomen Mittatekniikan keskuksen akkreditoima laboratorio T210).

Taulukko 1. Hankkeessa käytettävän metodiikan antama informaatio hemodynamiikasta.

**Finapress™**	**Colin 508T™**	**CircMon™**	**SphygmoCor PVMx™**
Pletysmografinen jatkuva verenpaine sormista	Tonometrinen jatkuva verenpaine arteria radialiksesta	Sydämen iskuvolyymi + jakautumisindeksi	Aorttatason verenpaine arteria radialiksen signaalin avulla
	Tonometrisen paineen autom. kalibrointi mansetilla olkavarresta	Minuuttivolyymi	Perifeerisen paineheijastuksen vaikutus sentraaliseen paineeseen
		Solunulkoinen nestemäärä	Subendokardiaalikudoksen toimintakykyisyys (viability)
		Vas. kammion työ	Vas. kammion ejektioaika
		Pulssiaallon etenemisnopeus	Pulssiaallon etenemisnopeus
		Valtimokomplianssi	Valtimokomplianssi
		Hengitystaajuus	
		Verenkierron ääreisvastus	

4.2. Veri- ja virtsanäytteet, DNA näytteet ja analyysit

DYNAMIC-tutkimukseen kuuluvat kattavat perus- ja erikoislaboratoriomääritykset (PVK, P-hCRP, P-Na, K, P-Ca, P-Krea, P-Uraat, fP-Gluc, P-ALAT, P-AFOS, fP-Kol, fP-HDL, fP-Trigly, fP-LDL, U-Kemseul, cU-alb, P-Kys-C, dU-Na, dU-K, varastoplasma ja -virtsa) sekä DNA näytteet. DNA-määritykset suoritetaan TAYS:n Laboratoriokeskuksen kliinisen kemian ja TaY:n oikeuslääketieteen yksiköissä (laitteistona ABI 7900HT, Applied Biosystems; DNA:n eristys- sekä pipetointirobotti Tecan Evo100). DNA-näytteenotto liittyy DYNAMIC-tutkimuksen protokollaan. DNA-näytteestä tutkitaan geneettisiä ominaisuuksia, joilla on merkitystä hemodynamiikan, nestetasapainon ja pulssiaallon etenemisnopeuden säätelyssä, valtimokovettumataudin synnyssä, sydämen ja verisuonten sähköisessä toiminnassa, verenpaineen ja rasva-aineenvaihdunnan säätelyssä, ja muiden valtimosairauksien vaaratekijöiden ilmentymisessä. Tätä tutkimusta varten määritetään lisäksi ennen lakritsialtistusta P-Renin, P-Aldos, dU-Aldos ja viikko altistuksen alusta sekä altistuksen lopussa P-K, P-Na, P-Krea, P-Renin, P-Aldos, dU-Na, dU-K, dU-Aldos, dU-kortisoli, dU-kortisoni, ja näiden tetrahydrometaboliittien eritys.

4.3 Lakritsialtistus

Ensimmäisten hemodynaamisten mittauksien jälkeen koehenkilöt nauttivat vähintään 60 g ja enintään 300 g lakritsia päivässä valmisteen glykyrritsiinipitoisuudesta riippuen (maksimi glykyrritsiiniannos 400 mg vuorokaudessa) korkeintaan 4 viikon ajan. Viikko lakritsialtistuksen alusta suoritetaan uudelleen hemodynaamiset mittaukset sekä hormonaaliset määritykset ja nämä samat tutkimukset toistetaan vielä lakritsialtistuksen lopussa. Tarkoituksena on, että koehenkilö nauttisi viimeisen lakritsiannoksensa mittauspäivänä. Halutessaan koehenkilö voi keskeyttää lakritsin syönnin milloin vain, mutta tällöin hänen toivotaan ottavan yhteyttä tutkijalääkäriin tai tutkimushoitajaan, jotta hemodynamiikan rekisteröinnit voidaan suorittaa mahdollisimman nopeasti lakritsin syönnin päätyttyä. Käytettävät lakritsimakeiset olivat mukana Elintarvikeviraston vuonna 1993 tehdyssä tutkimuksessa ja niiden glykyrritsiinipitoisuudeksi määriteltiin 0.096% (Kouvolan lakritsi) ja 0.31% (Halvan lakritsi) [11]. Valmistajien ilmoituksen mukaan lakritsimakeisten valmistusresepti ei ole muuttunut 1990-luvun alun jälkeen.

5. Toteutus

5.1 Tutkimushenkilöiden valinta

Tutkimushenkilöiksi valitaan terveitä normotensiivisiä vapaaehtoisia, jotka ovat valmiita nauttimaan päivittäin annoksen lakritsia 4 viikon ajan. Tutkimushenkilöiltä tarkistetaan ennen tutkimukseen hyväksymistä suullisesti aiemmat lakritsinkäyttötottumukset ja jos henkilö osoittautuu lakritsin suurkuluttajaksi (> 300 g lakritsia/viikko), ei häntä oteta mukaan tutkimukseen. Lisäksi tutkimukseen osallistujilta edellytetään 3 viikon täydellistä pidättäytymistä lakritsin ja lakritsijuuriuutetta sisältävien valmisteiden käytöstä ennen ensimmäistä hemodynaamista mittausta, jotta lähtötilanne saadaan luotettavasti mitattua.

5.2 Tutkimushenkilöiden poissulku tutkimuksesta

Tutkimukseen ei oteta mukaan raskaana olevia henkilöitä. Myös mikä tahansa sydän- ja verenkiertoelimistöön vaikuttava sairaus (esim. hypertensio, diabetes, sydänsairaudet, endokriiniset sairaudet, munuaissairaudet) tai säännöllinen lääkitys on esteenä tutkimukseen osallistumiselle.

5.3 Tutkimuksen kulku

Tutkimuspotilaat kutsutaan hemodynaamisten mittausten lisäksi vastaanottokäynnille, jonka aikana tutkijalääkäri haastattelee potilaat ja kerää perustiedot (sukupuoli, ikä, pituus, paino, vyötärönympärys, lantionympärys). Lääkitys, aikaisemmat sairaudet, verenpainehistoria, lipidihistoria, suku-, muuttohistoria, maitotuotteiden päivittäinen käyttö, liikuntatottumukset sekä tupakointi ja alkoholin käyttö kirjataan. Potilaita informoidaan tutkimuksesta ja heiltä pyydetään suostumus sekä tutkimukseen osallistumisesta että DNA-näytteen ottamisesta geneettistä analyysia varten. Vastaanottopäivänä tai myöhemmin sovittavana ajankohtana koehenkilöistä otetaan peruslaboratoriomääritykset, jonka jälkeen lakritsialtistus voidaan aloittaa. Ensimmäisen hemodynaamisen mittauksen yhteydessä tutkimushenkilöt saavat mukaansa päiväannospusseihin pakatun lakritsin viikon ajaksi. Toinen hemodynaaminen mittaus tehdään viikko lakritsialtistuksen alusta ja samalla otetaan uudelleen nestetasapainoon ja hormonitoimintaan liittyviä veri- ja virtsakokeita. Kolmannen kerran hemodynamiikkaa mitataan lakritsialtistuksen lopussa ja myös tällöin otetaan samat edellä mainitut veri- ja virtsakokeet.

Tietoja koskien koehenkilöiden terveyttä ja aiempien fysikaalisten mittausten tuloksia (verenpaine, paino, pituus, vyötärö/lantio mitat, silmäpohjat, ym.) kerätään myös potilastiedoista (esim. aiemmat sairaudet, lääkitykset). Hemodynaamiset mittaukset suorittaa koulutettu tutkimushoitaja TAYS:n kliinisen fysiologian yksikössä. Ennen mittauksia potilaaseen kiinnitetään tarvittavat elektrodit ja mittausanturit, jotka vastaavat sydänfilmin otossa ja verenpaineen mittaamisessa käytettäviä välineitä. Potilas asetetaan kallistettavalle tutkimuspöydälle, joka TILT-kokeessa nostetaan vaaka-asennosta 60° asteen kulmaan. Tutkimusprotokollan tarkempi kuvaus on alla olevassa taulukossa (taulukko 3.) ja sen läpivientiin kuluu noin 2 tuntia / koehenkilö. Tutkimuslääkkeet säilytetään KLF:n osastolla lukitussa kaapissa huoneessa, jossa suoritetaan lämpötilaseuranta.

Taulukko 3. Nitroglyseriinin ja salbutamolin vaikutus hemodynamiikkaan (kallistuskoe = TILT).

**NTG:n vaikutus hemodynamiikkaan**			**Salbutamolin vaikutus hemodynamiikkaan**		
Kokonaisaika	Mittaustapahtuma	Mittausaika	Kokonaisaika	Mittaustapahtuma	Mittausaika
0-5 min	Lepo	5 min	0-5 min	Lepo	5 min
5-10 min	TILT	5 min	5-10 min	TILT	5 min
10-15 min	Lepo	5 min	10-15 min	Lepo	5 min
15-20 min	Lepo / NTG 0.25 mg	5 min	15-20 min	Lepo / salbutamoli 400 µg (Volumatic)	5 min
20-25 min	TILT	5 min	20-25 min	TILT	5 min
25-30 min	Lepo	5 min	25-30 min	Lepo	5 min

Tutkimushoitaja tai laboratoriohoitaja ottaa joko hemodynaamisten mittausten yhteydessä tai erikseen sovittavana ajankohtana koehenkilöiltä verinäytteet erikoismäärityksiä (proteiinit, lipidit, kliiniskemialliset määritykset) ja DNA-määrityksiä varten, joka pakastetaan Tampereen yliopiston lääketieteen laitoksen laboratorion tiloihin (2 EDTA putkea, 1 sitraattiputki, 1 seerumiputki).

5.4 Seuranta lakritsialtistuksen aikana ja altistuksen keskeytys

Lakritsialtistuksen aikana tutkimushenkilöitä pyydetään seuraamaan painoaan päivittäin kotivaa'alla. Lakritsin mahdollisesti aiheuttamaa lievää verenpaineen nousua kontrolloidaan ensimmäisellä lakritsialtistusviikon jälkeen hemodynamiikan mittauksella sekä kahden altistusviikon jälkeen tutkimushoitajan tekemällä verenpaineen kaksoismittauksella. Mikäli verenpaine on normaalirajoissa, tutkimushenkilöt saavat mukaansa seuraavan lakritsiannoksen. Kontrollikäyntien yhteydessä tutkimushoitaja kyselee lakritsin syönnistä mahdollisesti aiheutuneita muita oireita ja ottaa tarvittaessa yhteyttä tutkijalääkäriin, jolloin harkitaan tapauskohtaisesti oirekuvan ja oireiden vaikeusasteen perusteella lakritsin syönnin keskeyttämistä potilasturvallisuuden säilyttämiseksi. Väliaikoina tutkimushenkilöitä neuvotaan ongelmien ilmaantuessa herkästi ottamaan yhteyttä tutkimushoitajaan tai tutkijalääkäriin puhelimitse.
Mikäli a) systolinen paine on noussut yli 30 mmHg lähtötasosta, b) diastolinen paine on noussut yli 15 mmHg lähtötasosta tai c) paino on noussut yli 3 kg, suoritetaan hemodynamiikan rekisteröinti ja näytteiden otto 3 vuorokauden kuluessa, minkä jälkeen lakritsialtistus keskeytetään.

5.5 Tiedonkeruu ja tutkimusrekisterin ylläpito

Tutkimushenkilöiden tiedot kerätään tiedonkeruulomakkeelle, jotka arkistoidaan kliinisen fysiologian tiloissa säilytettävään DYNAMIC tutkimuksen rekisteriin. Tutkimustiedot ja mittaustulokset tallennetaan sähköiseen muotoon, jossa yksittäiset tutkimuspotilaat esiintyvät koodinumeroina. Tutkimustietoihin pääsy on mahdollista vain tutkimusryhmän jäsenille sekä lääkelaitoksen tarkastajalle siinä määrin, mitä tietojen oikeellisuuden osoittaminen vaatii.

5.5 Tulosten julkaisuun ja tutkimussuunnitelman muutoksiin liittyvät asiat

Tutkimustulokset julkaistaan artikkelina lääketieteellisessä julkaisussa ja tämän artikkelin on tarkoitus olla pohjana väitöskirjatyölle. Mahdolliset puutteet tutkimussuunnitelmassa ja näistä aiheutuva muutostarve arvioidaan viipymättä. Mikäli tarpeen, tutkimussuunnitelmaan tehdään korjauksia. Kaikki tutkimussuunnitelman mahdolliset muutokset kirjataan, ja muutoksista lähetetään asianmukainen ilmoitus sekä PSHP:n eettiselle toimikunnalle että Lääkelaitokselle. Mikäli olennaisia muutoksia tehdään, tutkimusta ei jatketa ennen kuin eettinen toimikunta ja Lääkelaitos ovat muutokset hyväksyneet.

6. Eettiset näkökohdat

Lakritsi on elintarvike, jonka käytölle ei Suomessa ole asetettu rajoituksia. Myöskään glykyrritsiinille ei ole annettu käyttösuosituksia. Runsaasta lakritsin syönnistä voi aiheutua tutkimushenkilöille lähinnä ruuansulatuselimistön toiminnallisia vaivoja, mahdollisesti lievää painonnousua, lieviä neste- ja elektrolyyttitasapainon muutoksia sekä lievää verenpaineen nousua. Verenpainetta ja painoa seurataan tutkimuksen aikana tutkimushoitajan käynneillä, jotka sijoittuvat ensimmäiselle lakritsialtistusviikolle sekä toisen altistusviikon loppuun.

Päivittäiset glykyrritsiiniannokset jäävät alle 400 mg (2 kpl Halvan 60 gramman lakumattoa sisältää yhteensä 372 mg glykyrritsiiniä, 300 g Kouvolan lakritsia sisältää 288 mg glykyrritsiiniä,), eikä niistä pitäisi olla merkittävää haittaa terveille henkilöille. Glykyrritsiinin vaikutukset glukokortikoidimetaboliaan ovat palautuvia. Tutkimushenkilöillä on oikeus koska tahansa syytä ilmoittamatta keskeyttää lakritsinsyönti.

Tutkimusmenetelmä on kajoamaton eikä se aiheuta tutkimuspotilaalle kipua tai merkittävää haittaa. Hemodynaamisia muutoksia aiheuttamaan annettavat lääkkeet ovat pitkään markkinoilla olleita, turvallisia valmisteita ja tutkimuspotilaiden mahdolliset vasta-aiheet lääkkeiden annolle selvitetään etukäteen. Tutkimuksesta on jätetty ilmoitus Lääkelaitokselle. Nitroglyseriinin tavallisimmat haittavaikutukset ovat päänsärky, sydämen tykytys, verenpaineen lasku ja paikallinen kuumotus ja pistelyn tunne. Salbutamolin tavallisimmat haittavaikutukset ovat vapina, päänsärky ja sydämen tykytys. Mahdolliset haittavaikutukset ovat lyhytaikaisia ja ohimeneviä. Käytettävät lääkeannokset ovat normaalien kerta-annosten suuruisia, ja kumpaakin lääkeainetta annetaan tutkimuspotilaalle hemodynaamisen mittauksen yhteydessä vain kerran.

7. Lähdeviitteet

1. Elomaa, I., Lakritsioireyhtymä. *Duodecim*, 1975. 91(9): p. 600-6.
2. Sane, T., Lakritsi, aldosteroni ja verenpaine. *Duodecim*, 1994. 110(10): p. 974-80.
3. Sigurjonsdottir, H.A., K. Manhem, M. Axelson, and S. Wallerstedt, Subjects with essential hypertension are more sensitive to the inhibition of 11 beta-HSD by liquorice. *J Hum Hypertens*, 2003. 17(2): p. 125-31.
4. Enkovaara, A., Lakritsia lääkkeissä ja makeisissa. *Tabu*, 2003. 11(4): p. 11-12.
5. Stewart, P.M., A.M. Wallace, R. Valentino, D. Burt, C.H. Shackleton, and C.R. Edwards, Mineralocorticoid activity of liquorice: 11-beta-hydroxysteroid dehydrogenase deficiency comes of age. *Lancet*, 1987. 2(8563): p. 821-4.
6. Farese, R.V., Jr., E.G. Biglieri, C.H. Shackleton, I. Irony, and R. Gomez-Fontes, Licorice-induced hypermineralocorticoidism. *N Engl J Med*, 1991. 325(17): p. 1223-7.
7. Quinkler, M. and P.M. Stewart, Hypertension and the cortisol-cortisone shuttle. *J Clin Endocrinol Metab*, 2003. 88(6): p. 2384-92.
8. Heikens, J., E. Fliers, E. Endert, M. Ackermans, and G. van Montfrans, Liquorice-induced hypertension--a new understanding of an old disease: case report and brief review. *Neth J Med*, 1995. 47(5): p. 230-4.
9. Conn, J.W., D.R. Rovner, and E.L. Cohen, Licorice-induced pseudoaldosteronism. Hypertension, hypokalemia, aldosteronopenia, and suppressed plasma renin activity. *Jama*, 1968. 205(7): p. 492-6.
10. Olukoga, A. and D. Donaldson, Liquorice and its health implications. *J R Soc Health*, 2000. 120(2): p. 83-9.
11. Blomberg, K. and A. Hallikainen, Lakritsimakeisten glysyrritsiinipitoisuudet, in *Elintarvikeviraston julkaisuja* 10/1993, Helsinki.
12. Stormer, F.C., R. Reistad, and J. Alexander, Glycyrrhizic acid in liquorice--evaluation of health hazard. *Food Chem Toxicol*, 1993. 31(4): p. 303-12.
13. Magiakou, M.A., P. Smyrnaki, and G.P. Chrousos, Hypertension in Cushing's syndrome. *Best Pract Res Clin Endocrinol Metab*, 2006. 20(3): p. 467-82.
14. Suzuki, H., M. Handa, K. Kondo, and T. Saruta, Role of renin-angiotensin system in glucocorticoid hypertension in rats. *Am J Physiol*, 1982. 243(1): p. E48-51.
15. Saruta, T., Mechanism of glucocorticoid-induced hypertension. *Hypertens Res*, 1996. 19(1): p. 1-8.
16. Kelly, J.J., S.H. Tam, P.M. Williamson, J. Lawson, and J.A. Whitworth, The nitric oxide system and cortisol-induced hypertension in humans. *Clin Exp Pharmacol Physiol*, 1998. 25(11): p. 945-6.
17. Whitworth, J.A., M.A. Brown, J.J. Kelly, and P.M. Williamson, Mechanisms of cortisol-induced hypertension in humans. *Steroids*, 1995. 60(1): p. 76-80.
18. Stewart, P.M., Cortisol as a mineralocorticoid in human disease. *J Steroid Biochem Mol Biol*, 1999. 69(1-6): p. 403-8.
19. Kelly, J.J., G. Mangos, P.M. Williamson, and J.A. Whitworth, Cortisol and hypertension. *Clin Exp Pharmacol Physiol Suppl*, 1998. 25: p. S51-6.
20. Whitworth, J.A., G.J. Mangos, and J.J. Kelly, Cushing, cortisol, and cardiovascular disease. *Hypertension*, 2000. 36(5): p. 912-6.
21. Baid, S. and L.K. Nieman, Glucocorticoid excess and hypertension. *Curr Hypertens Rep*, 2004. 6(6): p. 493-9.
22. Capricchione, A., N. Winer, and J.R. Sowers, Adrenocortical hypertension. *Curr Hypertens Rep*, 2004. 6(3): p. 224-9.
