# Supplementary material for: Daily Liquorice Consumption for Two Weeks Increases Augmentation Index and Central Systolic and Diastolic Blood Pressure
Source: PLoS One. 2014 Aug 25;9(8):e105607. doi: 10.1371/journal.pone.0105607 (PMC4143270; doi:10.1371/journal.pone.0105607)
Supplement: Protocol S2 — English summary of trial protocol. (PDF) [file pone.0105607.s002.pdf]

PROTOCOL S2

The effect of liquorice intake on haemodynamics

# - parallel project of the DYNAMIC study

Investigators

Miia Leskinen, MD, University of Tampere
Ilkka Pörsti, Professor, Chief of Department, University of Tampere and Tampere University Hospital, Internal Medicine
Anna Tahvanainen, MD, University of Tampere
Jenni Koskela, MD, University of Tampere
Mika Kähönen, Professor, Chief of Department, University of Tampere and Tampere University Hospital, Clinical Physiology
Terho Lehtimäki, Professor, Chief Physician, University of Tampere and Tampere University Hospital, Clinical Chemistry
Tiit Kööbi, Docent, Chief Physician, Tampere University Hospital, Clinical Physiology
Jukka Mustonen, Professor, Chief Physician, University of Tampere and Tampere University Hospital, Internal Medicine

Principal Investigator

MD, PhD, Professor Ilkka Pörsti
School of Medicine, Department of Internal Medicine
FIN-33014 University of Tampere, Finland

Introduction

Regulation of blood pressure is a complex interplay between several haemodynamic mechanisms, and changes in for example arterial compliance, endothelial function, or the regulation of vascular resistance can be measured already before clinical manifestations of cardiovascular disease. Liquorice ingestion often elevates blood pressure, but the sensitivity of human subjects to the liquorice-induced elevation of blood pressure varies individually and patients with hypertension show higher sensitivity [1].

The liquorice-induced hypertension is mediated via mineralocorticoid receptor (MR) [2]. Liquorice contains glycyrrhizinic acid, which in the bowel is metabolized to glycyrrhetinic acid [3], which in turn inhibits the enzyme 11β-hydroxysteroid dehydrogenase type 2 and enhances the action of cortisol on the MR [4]. Excessive renal MR activation by cortisol promotes reabsorption of sodium from renal distal tubule and increases excretion of potassium and hydrogen into the urine [5]. This leads to sodium retention, increased extracellular volume, hypertension, hypokalemia and metabolic alkalosis. This condition is called pseudohyperaldosteronism due to low renin and low aldosterone concentrations in plasma, as described in 1968 by Conn [6]. The liquorice-induced hypertension is characterized by oedema, low plasma renin and aldosterone concentrations, but only in some patients hypokalemia [7].

The association of hypercortisolism and elevated blood pressure has been widely studied in Cushing's syndrome. Glucocorticoids cause hypertension through their intrinsic mineralocorticoid activity, through activation of the renin-angiotensin system, by enhancement of cardiovascular inotropic and pressor activity of vasoactive substances (catecholamines, vasopressin and angiotensin II), and by causing suppression of the vasodilatory systems (e.g. NO synthase) [8-11]. As a synergistic effect of the mechanisms described above, glucocorticoids increase cardiac output, total peripheral resistance and renovascular resistance, and elevate blood pressure [8].

Study objectives

Since the detailed hemodynamic effects underlying the liquorice-induced hypertension remain unknown, the objective of this study is to examine haemodynamic changes after regular liquorice exposure in healthy volunteers utilizing continuous pulse wave analysis, whole-body impedance cardiography, tonometric radial artery and plethysmographic finger BP recordings during passive orthostatic challenge. In addition, the study objective is to establish new tools to identify patients with secondary hypertension. Recognizing liquorice-induced hypertension is important since it can be treated with refraining from liquorice and thus unnecessary medications can be avoided.

Study subjects and methods

This study is a parallel project of the DYNAMIC study, which is registered in the EU Clinical Trials Register (EudraCT-number 2006-002065-39), and is approved by the Ethics Committee of Tampere University Hospital and by the Finnish Medicines Agency (study code number R06086M). Later the study was also registered in the ClinicalTrials.gov database (ID: NCT01742702).

Maximum number of 40 healthy, normotensive subjects will be recruited for the liquorice study. Exclusion criteria are office blood pressure over 140/90 mmHg, any cardiovascular disease with regular medication, or pregnancy, or consumption of liquorice over 300 g per week. The suitability of all participants for the study will be confirmed by medical and laboratory examinations.

The haemodynamic status of study subjects will be examined *before and after the liquorice exposure* utilizing the DYNAMIC study protocol using continuous pulse wave analysis, whole-body impedance cardiography, tonometric radial artery and plethysmographic finger BP recordings during passive head-up tilt. The method collects functional beat-to-beat data from about 50 different cardiovascular variables simultaneously, including peripheral and central BP, heart rate, cardiac output, systemic vascular resistance, pulse wave velocity, central wave reflections, and autonomic nervous tone.

The haemodynamic recordings are performed in the Unit of Clinical Physiology of Tampere University Hospital by trained research nurses. The patient is lying supine on the tilt table and the electrodes and equipment are connected, and an introductory tilt is performed by raising the tilt-table to about upright 60° angle. The actual protocol consists of repeated 5-min periods of recording: 5-min supine, 5-min upright, 5 min supine. Then the recording is repeated with 0.25 sublingual nitroglycerin. After a 60-min break, the whole protocol is repeated with 400 µg salbutamol as the test drug.

After the first haemodynamic measurements, voluntary subjects will consume daily 60 to 300 g of liquorice (depending on the glycyrrhizin concentration of the commercial liquorice product) for a maximum period of 4 weeks. The estimated daily dose of glycyrrhizin is 290-370 mg from Halva liquorice™ or Kouvola liquorice™ products. Comprehensive non-invasive haemodynamic recordings are repeated at the end of the liquorice ingestion period.

In the DYNAMIC study routine laboratory measurements include blood count, plasma C-reactive protein, sodium, potassium, calcium, creatinine, cystatin-C, glucose, liver enzymes, lipid profile, urine dipstick tests, microalbuminuria determination, and urine 24-hour sodium and potassium excretions. Also several samples are taken for special determinations (proteins, lipids, hormones, other determinations in clinical chemistry, and also DNA-samples from all subjects). In the liquorice study, laboratory measurements will include also plasma renin and aldosterone and urine aldosterone (before and after the liquorice exposure), and plasma potassium, sodium, creatinine, and urine sodium, potassium, cortisol, cortisone and tetrahydrometabolites of cortisol and cortisone (after the liquorice exposure).

Data management

The data will be recorded in specifically prepared forms and kept in a locked depository. All clinical data will be recorded into an electronic database where single study subjects exist as coded numbers and identification is not possible. Only the study personnel have access to the data. In addition, the inspector of the Finnish Medicines Agency (Fimea) is allowed to see research material as far as is needed to indicate the validity of the data.

Ethical considerations

Recording method of haemodynamics is non-invasive and does not cause pain or notable discomfort. The risks imposed upon the study subjects are small and have been adequately taken into consideration. Ethical approval has been obtained for DYNAMIC study from the Ethics Committee of Pirkanmaa Hospital District, and the study has also been approved by the Finnish Medicines Agency (Fimea).

Substantial amount of liquorice may cause mainly functional disorders of the digestive tract, possibly weight gain or slight changes in water and electrolyte equilibrium and slight elevation of blood pressure. If the daily glycyrrhizin dose exceeds 400 mg, the risk of adverse events increases [12]. In this study, the estimated daily dose of glycyrrhizin is 290-370 mg and these doses should not cause any significant harm to healthy subjects. The effects of glycyrrhizin to glucocorticoid metabolism are reversible. The participants are advised to inform the study group immediately if they gain weight over 3-4 kilograms, observe oedema in the lower extremities or encounter other problems during liquorice ingestion. The study subjects are entitled to discontinue the liquorice intervention anytime without declaring a reason.

References

1. Sigurjonsdottir HA, Manhem K, Axelson M, Wallerstedt S (2003) Subjects with essential hypertension are more sensitive to the inhibition of 11 β-HSD by liquorice. J Hum Hypertens 17: 125-131.
2. Frey FJ, Odermatt A, Frey BM (2004) Glucocorticoid-mediated mineralocorticoid receptor activation and hypertension. Curr Opin Nephrol Hypertens 13: 451-458.
3. Ploeger B, Mensinga T, Sips A, Seinen W, Meulenbelt J, et al. (2001) The pharmacokinetics of glycyrrhizic acid evaluated by physiologically based pharmacokinetic modeling. Drug Metab Rev 33: 125-147.
4. Stewart PM, Wallace AM, Valentino R, Burt D, Shackleton CH, et al. (1987) Mineralocorticoid activity of liquorice: 11-beta-hydroxysteroid dehydrogenase deficiency comes of age. Lancet 2: 821-824.
5. Edwards CR, Stewart PM, Burt D, Brett L, McIntyre MA, et al. (1988) Localisation of 11 beta-hydroxysteroid dehydrogenase--tissue specific protector of the mineralocorticoid receptor. Lancet 2: 986-989.
6. Conn JW, Rovner DR, Cohen EL (1968) Licorice-induced pseudoaldosteronism. Hypertension, hypokalemia, aldosteronopenia, and suppressed plasma renin activity. JAMA 205: 492-496.
7. Olukoga A, Donaldson D (2000) Liquorice and its health implications. J R Soc Promot Health 120: 83-89.
8. Magiakou MA, Smyrnaki P, Chrousos GP (2006) Hypertension in Cushing's syndrome. Best Pract Res Clin Endocrinol Metab 20: 467-482.
9. Suzuki H, Handa M, Kondo K, Saruta T (1982) Role of renin-angiotensin system in glucocorticoid hypertension in rats. Am J Physiol 243: E48-51.
10. Saruta T (1996) Mechanism of glucocorticoid-induced hypertension. Hypertens Res 19: 1-8.
11. Kelly JJ, Tam SH, Williamson PM, Lawson J, Whitworth JA (1998) The nitric oxide system and cortisol-induced hypertension in humans. Clin Exp Pharmacol Physiol 25: 945-946.
12. Stormer FC, Reistad R, Alexander J (1993) Glycyrrhizic acid in liquorice--evaluation of health hazard. Food Chem Toxicol 31: 303-312.
